# Analysis of Whole Exome Sequencing Results of Type 2 Diabetic Pedigree

**DOI:** 10.1002/mgg3.70296

**Published:** 2026-10-21

**Authors:** Jie Chen, Jia‐Li Li, He‐Jia Yin, Wei‐Chang Guo, Hui‐Fang Li

**Affiliations:** ^1^ The First Affiliated Hospital of Kunming Medical University Kunming Yunnan China; ^2^ Kunming Medical University Kunming Yunnan China; ^3^ Yunnan University Kunming Yunnan China; ^4^ Yunnan Key Laboratory of Laboratory Medicine Kunming Yunnan China

**Keywords:** EPHX3, FHOD1, gene mutation, genomic exon sequencing, type 2 diabetes mellitus

## Abstract

**Background:**

Type 2 diabetes mellitus (T2DM) exhibits strong familial aggregation. This study aimed to identify potential pathogenic genes and mutation sites in a Chinese pedigree with a high prevalence of T2DM.

**Methods:**

A multigenerational family with 20 members, including 5 diagnosed with T2DM, was enrolled. The proband was a second‐generation female with a 10‐year disease history. Peripheral blood was collected, genomic DNA was extracted from blood cells, and whole exome sequencing was performed. Bioinformatic analysis, including KEGG pathway mapping, was used to screen candidate pathogenic variants.

**Results:**

Sequencing revealed 485 DNA variants, which were filtered to 16 high‐priority mutations (15 SNPs and 1 Indel). Through literature review and functional pathway analysis, two heterozygous mutations—EPHX3 rs760409070 [c.778G>A, p.Glu260Lys] and FHOD1 rs142023201 [c.1493A>G, p.Asn498Ser]—were identified as candidate pathogenic variants. These two mutations were consistently present in all affected family members.

**Conclusion:**

The EPHX3 rs760409070 and FHOD1 rs142023201 mutations are likely contributing genetic factors to T2DM in this pedigree. Based on bioinformatic analysis, we hypothesize that these mutations may promote diabetes pathogenesis by reducing peripheral insulin sensitivity, impairing glucose uptake, suppressing glycogen synthesis, and enhancing gluconeogenesis.

## Graphical abstract

Mutations of EPHX3 gene rs760409070[c.778 G>A (p.Glu260Lys)] and FHOD1 gene rs142023201 [c.1493 A>G (p.Asn498Ser)] are likely contributing genetic factors to T2DM in this pedigree. Based on bioinformatic analysis, we hypothesize that these mutations may promote diabetes pathogenesis by reducing peripheral insulin sensitivity, impairing glucose uptake, suppressing glycogen synthesis, and enhancing gluconeogenesis.

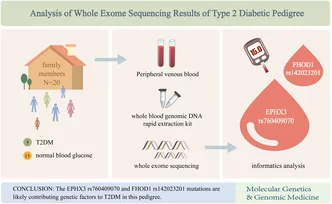

## 1 Introduction

Diabetes mellitus (DM) is a complex disease caused by environmental and genetic factors. It is accompanied by insufficient insulin secretion and/or insulin resistance, characterized by chronic hyperglycemia, chronic damage, dysfunction and even multiple organ failure. The global prevalence of DM in adults aged 20–79 years was 10.5% in 2021, which is expected to increase to 12.2% by 2045 (Sun et al. 2022). In the DM population, 90% are type 2 diabetes mellitus (T2DM) patients. The specific pathogenesis of T2DM has not yet been clarified. It is generally suggested that genetic factors play an important role in the pathogenesis of T2DM, and there are hundreds of T2DM susceptibility genes identified by Genome‐wide association study (GWAS) (Suzuki et al. 2024). As reported by several studies in China (Liao et al. 2023; Tam et al. 2014; Wei et al. 2010), the first‐degree relatives with normal blood glucose in T2DM families are associated with a potential risk of diabetes; moreover, studies in Thailand (Tangjittipokin et al. 2025), Maryland (Kalyani et al. 2025), Sub‐Saharan Africa (Asamoah et al. 2020), and Moroccan (Benrahma et al. 2011) also suggest a certain familial aggregation in the occurrence of T2DM. Therefore, it is significant to screen pathogenic genes and mutation sites in T2DM families, so as to prevent, diagnose and treat T2DM.

At present, most of the mutations of DM virulence genes have been identified to be base mutations in the exon region (Shojima and Yamauchi 2023; Walker et al. 2023), most of which are low frequency and rare mutations. Whole exome sequencing is a genomic analysis method which uses sequence capture technology to capture and enrich DNA in the whole genome exon region for high‐throughput sequencing. It is highly sensitive to low frequency and rare mutations, and can detect most disease‐related mutations in the exon region (Halldorsson et al. 2022). Currently, whole exome sequencing has been regarded as the most promising research method in the molecular mechanism research and molecular diagnosis of complex diseases such as DM (Huerta‐Chagoya et al. 2024).

Due to the genetic heterogeneity of T2DM, the major pathogenic genes and mutation sites may vary among different races or different families of the same race (Deutsch and Udler 2025). At present, no relevant studies have been conducted to search the pathogenic genes and mutation sites in T2DM families in Yunnan Province of China by whole exome sequencing technology. Therefore, this study aimed to identify possible high‐impact genetic variants contributing to the strong familial aggregation of T2DM in a Chinese pedigree using whole exome sequencing and bioinformatic analysis, so as to provide clues and theoretical basis for the prevention, diagnosis, and treatment of familial type 2 diabetes.

## 2 Materials and Methods

### 2.1 Editorial Policies and Ethical Considerations

This study was approved by the Institutional Review Board of The First Affiliated Hospital of Kunming Medical University (Approval No. 2020‐L‐18), and conducted in accordance with the Declaration of Helsinki. All the family members provided written informed consent and approved this study.

### 2.2 Study Design and Patients

Proband: The patient was a 55‐year‐old woman who met the 1999 World Health Organization diagnostic criteria for diabetes, including the presence of diabetic symptoms (typical symptoms such as polyuria, polydipsia, polyphagia, and unexplained weight loss) plus random blood glucose ≥ 11.1 mmol/L, or fasting blood glucose (FBG) ≥ 7.0 mmol/L, or 2‐h postprandial blood glucose (PBG) ≥ 11.1 mmol/L in an oral glucose tolerance test. The proband had a 10‐year history of DM and was diagnosed with T2DM based on the characteristics of her medical history and laboratory findings.

Family members: There were 20 members in this family, among whom the proband's mother in the first generation (G1) and the proband's sister in the second generation (G4, G5, G7) were all patients with T2DM. The blood glucose levels of the second generation family members, including brother of the proband (G9), husband of the proband (G3), brother‐in‐law of the proband (G6, G8), sister‐in‐law of the proband (G10), and all members of the third generation, did not exhibit any obvious abnormality. There was no history of inbreeding or other hereditary diseases in the family (Figure 1). The clinical features of the five affected members (G1, G2, G4, G5, G7) are summarized in Table S1. The mean age at diagnosis was 43.2 years, and all affected individuals were overweight or obese (body mass index [BMI] ≥ 25 kg/m^2^). Three patients were treated with metformin‐based regimens, while two required insulin therapy. Diabetic peripheral neuropathy was present in four patients, and the eldest patient (G1) had both diabetic nephropathy and retinopathy.

**FIGURE 1:**
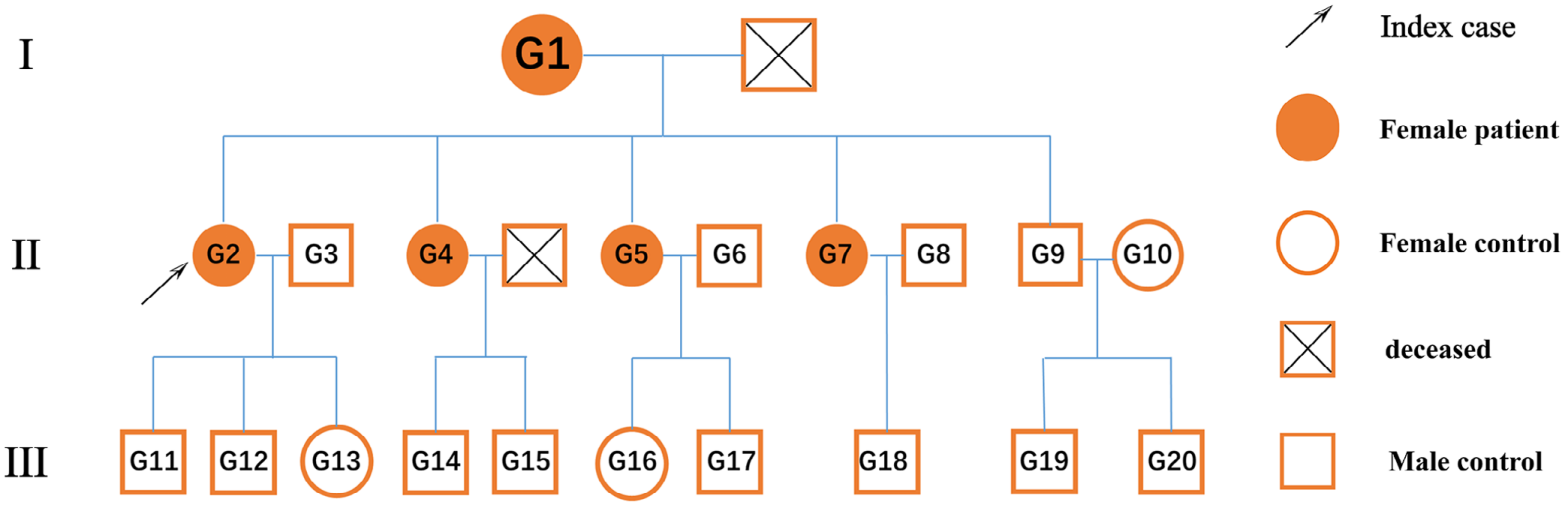
A family with T2DM pedigree. I, II and III indicate the 1st, 2nd and 3rd generations, respectively.

### 2.3 Whole Blood Genomic DNA Extraction

Fasting peripheral venous blood (4 mL) was collected from each of the 20 family members into an anticoagulant tube, and genomic DNA samples were extracted from peripheral blood using the Rapid Blood DNA Kit (Beijing Biomed Gene Technology Co. Ltd., Beijing, China; Catalog No. DL101‐01) according to the manufacturer's instructions. DNA concentration was measured using the Qubit dsDNA Assay Kit (Invitrogen, Beijing, China; Catalog No. Q32854) with a Qubit fluorometer, and DNA integrity was evaluated by 1% agarose gel electrophoresis with λ‐Hind III digest DNA Marker (Takara Bio, Beijing, China; Catalog No. 3403) and D2000 DNA Marker (Tiangen Biotech (Beijing) Co. Ltd., Beijing, China; Catalog No. MD114).

### 2.4 Whole Exon Sequencing

DNA samples extracted from 20 family members were then sent to Shenzhen BGI Co. Ltd. (Shenzhen, China) for whole exome sequencing using the BGISEQ‐500 sequencing system. Library preparation and exome capture were performed using the SureSelect Human All Exon V6 kit (Agilent Technologies, Santa Clara, CA, USA; Catalog No. 5190‐8863), and DNA fragment purification was conducted using the Agencourt AMPure XP‐Medium kit (Beckman Coulter Inc., Shanghai, China; Catalog No. A6388) according to the manufacturer's protocols.

### 2.5 In Silico Prediction Methods for Single Nucleotide Polymorphisms (SNPs) Analysis

#### 2.5.1 Bioinformatic Analysis With Significance Analysis of SNP/Indel Site

For any mutation, the mutation frequency Pv in a family can be calculated by the following formula: Pv = *p* ^m^ (1 − *p*)^nm^. Where, *p* represents the mutation frequency of this mutation in the normal population minor allele frequency (MAF) (0 < *p* < 1), *n* is the total number of samples, *m* indicates the number of samples with this mutation observed, and Pv is indicative of the probability of *m* mutations observed in *n* samples. In this study, genetic information in the family was obtained; as a result, only 5 diabetic patients in the family carried mutations, while the control samples did not harbor mutations, and the occurrence frequency in the third generation of immediate family members was no more than 50% (5 times), in other words, *m* = 5 and *n* = 20. The mutation frequency of the normal population at each site was derived from thousand genome data (1000G) (Lek et al. 2016) and exome integration database (ExAC) (Karczewski et al. 2017). Some SNPs did not have available mutation frequency information in these two groups of data, so the rsid of SNPs was used to query in dbSNP, and the maximum MAF values of these sites were then obtained from the database (Sherry et al. 2001). As corresponding MAF values for the brand new SNP sites discovered in this project were unavailable in the three databases 1000G, ExAC, and dbSNP, a relatively small MAF value was set for the Manhattan map and subsequent analysis. In this study, the minimum MAF value was set at 1e‐4.

#### 2.5.2 Bioinformatic Analysis With Manhattan Map

To obtain SNP and Indel mutation sites specific to this T2DM family, the Manhattan map was employed to analyze and display the information of SNP and Indel sites. In brief: (1) the exon DNA mutation probability formula of family members was utilized to calculate the Pv values corresponding to each SNP site and Indel site. (2) The Manhattan function in “qqman” (Montojo et al. 2010) of R software package was adopted to display SNP and Indel information of this T2DM family. (3) The threshold of display rs number in the Manhattan map was set at Pv = 1e‐9. (4) Due to the large number of SNP and Indel sites, the Manhattan map was divided into showing all SNP/Indel sites and showing the SNP/Indel sites with the function of changing proteins and MAF < 0.001 only, so as to obtain more clear biological significance of SNP sites and Indel sites.

#### 2.5.3 Bioinformatic Analysis With Specific SNP/Indel Proteins and Potential Mechanisms Analysis

The genemania (Lin et al. 2010) was applied in locating genes corresponding to all mutation sites in the above results in the Kyoto Encyclopedia of Genes and Genomes (KEGG) pathway (Kanehisa et al. 2023), and then gene interaction analysis was conducted. Finally, the possible pathogenic genes and mutation sites in this T2DM family were screened, and the mechanisms of these mutation sites in affecting and inducing the occurrence and development of DM were explored.

### 2.6 In Silico Functional Prediction and Database Annotation of Missense Variants

The potential functional consequences of the identified missense variants were assessed using the Ensembl Variant Effect Predictor (VEP), including SIFT (Sorting Intolerant From Tolerant), PolyPhen‐2 (Polymorphism Phenotyping v2), CADD (Combined Annotation Dependent Depletion), and REVEL (Rare Exome Variant Ensemble Learner). SIFT scores ≤ 0.05 were considered damaging; PolyPhen‐2 scores > 0.909 as probably damaging, 0.447–0.909 as possibly damaging, and ≤ 0.447 as benign; CADD PHRED scores ≥ 20 as deleterious; and REVEL scores > 0.75 as damaging. The MANE Select transcript was used as the primary reference for each gene (ENST00000221730.8 for EPHX3 and ENST00000258201.9 for FHOD1).

In addition, we cross‐referenced the two candidate variants with public genomic and phenotype databases to assess allele frequencies and prior phenotype associations. Allele frequency data were obtained from the Genome Aggregation Database (gnomAD v4.1.1). Clinical significance classifications were retrieved from ClinVar. Phenotype associations were queried using the GWAS Catalog (accessed June 2026) and Ensembl phenotype data (release 116).

### 2.7 Statistical Analysis

Bioinformatic statistical analysis was carried out using R software.

## 3 Results

### 3.1 DNA Sample Quality Assessment

Quality assessment of 20 DNA samples: On the one hand, Qubit was employed to detect the concentration and calculate the total amount of DNA, with ≥ 1 μg being considered as the standard. On the other hand, DNA integrity was detected by 1% agarose gel electrophoresis (AGE).

### 3.2 Exon Sequencing Quality

Genome wide exon sequencing was performed on 20 DNA samples, and the average number of original bases was 14,890.66 Mb per sample. After removing low‐quality reads, an average of 148,819,404 clean reads (14,873.84 Mb) were obtained per sample, with an average GC content of 52.14%. Moreover, the clean reads of each sample had high Q20 and Q30, indicating that the sequencing data were of high sequencing quality (Table 1).

**TABLE 1:** Statistical table of exon sequencing data from 20 family members.

Samples	Raw reads	Raw bases (Mb)	Clean reads	Clean bases (Mb)	Clean data rate (%)	Clean read1 Q20 (%)	Clean read2 Q20 (%)	Clean read1 Q30 (%)	Clean read2 Q30 (%)	GC content (%)
G1	131,119,440	13,811.94	137,908,684	13,781.51	99.78	97.88	95.95	91.81	87.65	50.81
G2	130,394,084	13,039.41	130,207,926	13,014.61	99.81	97.72	95.9	91.23	87.5	51.62
G3	132,424,844	13,242.48	132,242,176	13,216.94	99.81	97.72	96.52	91.31	88.99	51.56
G4	143,256,438	14,325.64	143,056,346	14,299.49	99.82	97.7	96.06	91.26	88.01	52.7
G5	153,148,540	15,314.85	152,934,992	15,279.37	99.77	97.87	96.34	91.8	88.57	51.38
G6	155,506,998	15,550.7	155,289,170	15,511.56	99.75	97.93	96.6	92	89.32	50.67
G7	153,361,172	15,336.12	153,316,202	15,319.47	99.89	97.63	96.78	90.92	89.44	51.59
G8	124,339,274	12,433.93	124,299,334	12,423.1	99.91	97.99	96.57	92.01	89.03	52.53
G9	156,717,562	15,671.76	156,686,430	15,660.46	99.93	97.87	96.26	91.57	88.2	52.96
G10	154,783,670	15,478.37	154,747,200	15,469.8	99.94	97.84	96.15	91.5	87.91	52.92
G11	131,705,054	13,170.51	131,651,396	13,160.87	99.93	97.79	95.94	91.24	87.35	52.89
G12	155,099,254	15,509.93	155,064,004	15,500.51	99.94	97.84	96.35	91.48	88.52	52.72
G13	164,963,336	16,496.33	164,928,054	16,482.93	99.92	97.85	96.31	91.58	88.45	52.98
G14	157,648,864	15,764.89	157,613,958	15,752.78	99.92	97.94	96.66	91.86	89.38	53.03
G15	154,481,096	15,448.11	154,435,136	15,432.12	99.9	97.87	97.87	91.53	88.42	50.41
G16	155,078,752	15,507.88	155,045,094	15,497.22	99.93	97.95	91.91	89.65	50.74	99.93
G17	141,156,208	14,115.62	141,120,236	14,108.24	99.95	97.75	95.71	91.2	86.8	52.09
G18	164,554,102	16,455.41	164,514,380	16,444.42	99.93	97.88	95.8	91.58	87.15	52.95
G19	148,476,624	14,847.66	148,446,062	14,839.55	99.95	97.89	97.89	91.65	89.13	53.35
G20	162,917,508	16,291.75	162,881,312	16,281.93	99.94	97.81	96.32	91.09	88.36	52.89

*Note:* (1) Raw reads: number of original sequencing sequences; (2) raw bases: number of original bases; (3) clean reads: sequence number of sequencing data obtained after preliminary filtering; (4) clean bases: number of bases obtained after removing low‐quality bases; (5) clean read1 and clean read2: reads obtained by double‐ended sequencing. The software gives a mass value for each base, which represents the correct identification rate of the sequencing base; meanwhile, 20 represents the normal identification rate of the base 99%, 30 represents the correct identification rate of the base 99.9%, Q20 (%) indicates the percentage of bases with mass value ≥ 20; and Q30 (%) suggests the percentage of bases with mass value ≥ 30. Q20 and Q30 were both greater than 80%, indicating good sequencing quality and normal follow‐up analysis.

### 3.3 Statistical Results of Mutation Detection

#### 3.3.1 SNP Detection Results

An average of 96,114 SNP mutation sites were detected in all samples. There were 11,002 synonymous mutations and 10,381 missense mutations in the coding region. In the meantime, 32 SNP sites that changed the stop codon to non‐stop codon, 94 SNP sites that changed the non‐stop codon to stop codon, 19 SNP sites that changed the start codon to non‐start codon, and 93 SNP sites that changed the splice receptor or splice donor in the splice site region were also discovered.

#### 3.3.2 Indel Detection Results

An average of 15,142 Indel mutation sites were obtained in all the samples. Meanwhile, 308 frameshift mutations were detected in the coding region; 0 mutations changed the stop codon to non‐stop codon or the start codon to non‐start codon, and 93 mutations changed the splicing receptor or splicing donor in the splicing site region.

### 3.4 Bioinformatic Analysis

Through whole exome sequencing, sites existing in all the T2DM patients but did not have mutations in normal controls were selected. Considering that the third generation of family members also had a potential risk of disease, the occurrence frequency of SNP and Indel sites in the third generation of immediate family members was further required to be no more than 50% (≤ 5 times). Finally, 485 mutation sites meeting the requirements were screened, including 429 SNP sites and 56 Indel sites.

After calculating the significance of each SNP site and Indel site, a Manhattan map was adopted for display. In Figure 2A, the qqman software package was employed to perform a Manhattan map display for all SNP sites, and the blue line and red line thresholds were set by qqman default. As observed from Figure 2A, there were lots of significant SNPS on the whole (more than 252). After the snp effect software was utilized to annotate these SNP sites (Montojo et al. 2010), 39 SNP sites with moderate to high biological function influence were discovered. After removing SNP sites with MAF ≥ 0.001, 15 significant SNP mutation sites were finally identified (Figure 2B). Using the same method, a total of 32 significant Indel sites and 1 Indel mutation with medium to high biological function influence were detected (Figure 3).

**FIGURE 2:**
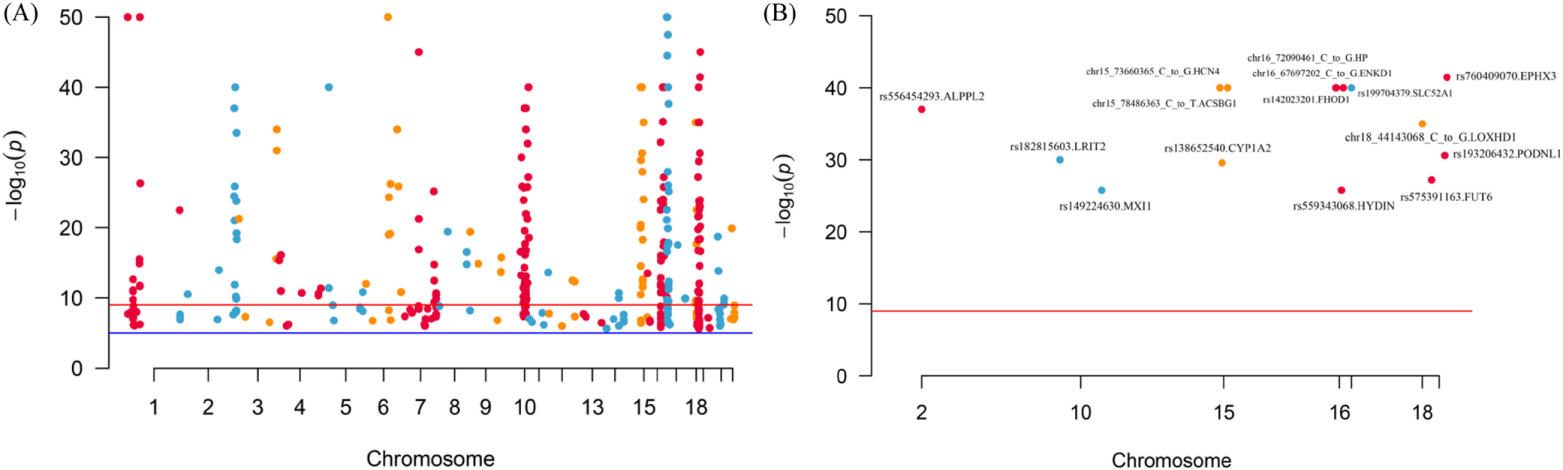
Manhattan map displaying all the SNP sites (A) and Manhattan map showing the significant SNP mutation sites (B). (1) *X*‐axis shows the position of the chromosome where SNP is located. (2) *Y*‐axis is the −log_10_ (*p*) value corresponding to each SNP site. The greater −log_10_ (*p*) value indicates the stronger association between this site and the disease. (3) Points on the blue line represent significant SNP sites (threshold = 1e‐5), and those above the red line represent extremely significant SNP sites (threshold = 1e‐9). (4) In figure B, the positions of rs142023201.FHOD1 sites and chr16_67,697,202_C_to_G.ENKD1 sites overlapped in the Manhattan map because of the same position on chromosomes and the same −log_10_ (*p*) value.

**FIGURE 3:**
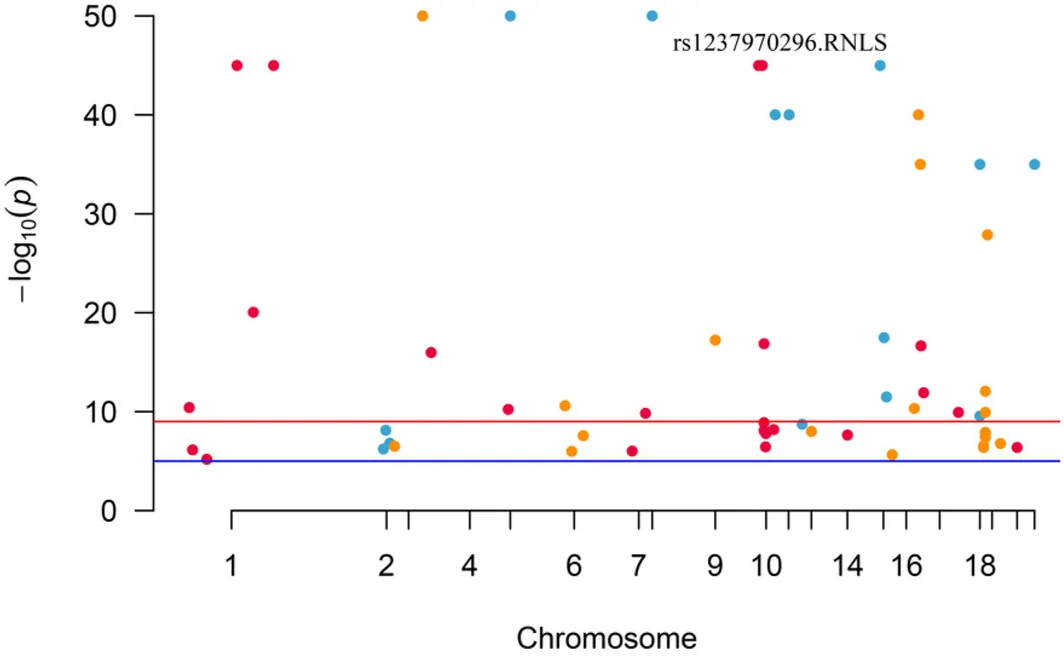
Manhattan map displaying Indel mutation sites. (1) *X*‐axis is the position of chromosome where insertion or deletion is located. (2) *Y*‐axis indicates the −log_10_ (*p*) value corresponding to each insertion or deletion site. (3) The blue horizontal line represents the background baseline of *p* = 1e‐5, and the red horizontal line stands for the baseline moderate significance of *p* = 1e‐9. (4) The insertion or deletion sites (rs1237970296.RNLS) changing functions of proteins in the figure were labeled in the risd.geneid format.

In summary, 15 significant SNP mutation sites and 1 Indel mutation site in this family were eventually obtained (Table 2). These mutations were found in 16 genes, including EPHX3, SLC52A1, HCN4, ACSBG1, FHOD1, ENKD1, HP, ALPPL2, LOXHD1, PODNL1, LRIT2, CYP1A2, FUT6, MXI1, HYDIN, and RNLS. Among them, the mutations in five genes, HCN4, ACSBG1, ENKD1, HP, and LOXHD1, were unreported mutations, including chr15_73,660,365_C_to_G.HCN4, chr15_78,486,363_C_to_T.ACSBG1, chr16_67,697,202_C_to_G.ENKD1, chr16_72,090,461_C_to_G.HP, and chr18_44,143,068_C_to_G.LOXHD1.

**TABLE 2:** List of significant mutation sites in pedigree.

SNP/Indel	CHR	BP	*p*	Function	Gene	1000G	ExAC
rs760409070.EPHX3	19	15,338,661	3.54769805952716E‐42	missense_variant	EPHX3	0.0000248	0.0000248
rs199704379.SLC52A1	17	4,937,014	9.99000449880022E‐41	missense_variant	SLC52A1	0.0001	0.0001
chr15_73,660,365_C_to_G.HCN4	15	73,660,365	9.99880006599781E‐41	missense_variant	HCN4	NA	NA
chr15_78,486,363_C_to_T.ACSBG1	15	78,486,363	9.99880006599781E‐41	splice_acceptor_variant+intron_variant	ACSBG1	NA	NA
rs142023201.FHOD1	16	67,268,280	9.99880006599781E‐41	missense_variant	FHOD1	0	0
chr16_67,697,202_C_to_G.ENKD1	16	67,697,202	9.99880006599781E‐41	missense_variant	ENKD1	NA	NA
chr16_72,090,461_C_to_G.HP	16	72,090,461	9.99880006599781E‐41	missense_variant	HP	NA	NA
rs556454293.ALPPL2	2	233,271,626	1.0057733856828E‐37	missense_variant	ALPPL2	0.000199681	0.0001
chr18_44,143,068_C_to_G.LOXHD1	18	44,143,068	9.99870007799715E‐36	missense_variant	LOXHD1	NA	NA
rs193206432.PODNL1	19	14,044,041	2.57266143377517E‐31	missense_variant	PODNL1	0.000399361	0.0003
rs182815603.LRIT2	10	85,982,013	9.74362582809287E‐31	missense_variant	LRIT2	0.000998403	0.0002
rs138652540.CYP1A2	15	75,042,327	2.5214664586159E‐30	missense_variant	CYP1A2	0.000199681	0.0001
rs575391163.FUT6	19	5,831,524	6.43937192948815E‐28	missense_variant	FUT6	0.000399361	0.0001
rs149224630.MXI1	10	112,044,663	1.64639985217912E‐26	missense_variant	MXI1	0.000599042	0.0007
rs559343068.HYDIN	16	70,894,075	1.64639985217912E‐26	missense_variant	HYDIN	0.000599042	0.0001
indel_rs1237970296.RNLS	10	90,342,872	9.99890005499836E‐46	frameshift_variant	RNLS	NA	NA

*Note:* CHR: the chromosome where the SNP site is located; BP: the chromosome location of SNP; *p* value: *p*‐value corresponding to the SNP; Gene: indicates the Gene name corresponding to the SNP. 1000G and ExAC refer to the mutation frequencies in 1000G and ExAC databases, respectively. The last line indicates the information for Indel.

Abbreviation: SNP, single nucleotide polymorphism.

### 3.5 Genes Related to Diabetes

By reviewing relevant literature and comparing these 16 genes with KEGG pathway, EPHX3 and FHOD1 genes were eventually screened to be related to T2DM development in this family (Alaaeldin et al. 2021; He et al. 2023; Kwak and Park 2016; Murthy et al. 2000; Teaney and Cyr 2023; Tojo et al. 2003; Xu et al. 2022).

#### 3.5.1 EPHX3 Gene rs760409070 Site Mutation

There are complex genetic interactions between EPHX3 and phosphatidylinositol‐4,5‐bisphosphate 3‐kinase catalytic subunit beta (PIK3CB) genes in the KEGG pathway related to DM pathogenesis. Phosphoinositide 3‐kinase (PI3K), the PIK3CB gene encoding product, is an important member of the phospholipase family, which is also an important conduction molecule involved in various signal transduction pathways in the body. In the KEGG pathway diagram, PI3K formed the PI3K/protein kinase B (AKT/PKB) signaling pathway with downstream signal molecule AKT (Figure 4), which promoted the ability of Glucose transporter 4 (GLUT4) in the uptake and transport of glucose, enhanced the activity of Glycogen Synthetase Kinase‐3 (GSK‐3) to promote glycogen synthesis, and promoted the inhibition of Forkhead box transcription factor O1 (FOXO1) on gluconeogenesis, thereby reducing the plasma glucose level. The mutation of EPHX3 gene led to the inhibition of PI3K/AKT signaling pathway on GLUT4 for the uptake and transport of glucose, on GSK‐3 for the promotion of glycogen synthesis, and on FOXO1 for the inhibition of gluconeogenesis. As a result, it led to insulin resistance, inhibited glucose uptake and transport, suppressed glycogen synthesis, and promoted gluconeogenesis, finally resulting in the occurrence of T2DM in this family.

**FIGURE 4:**
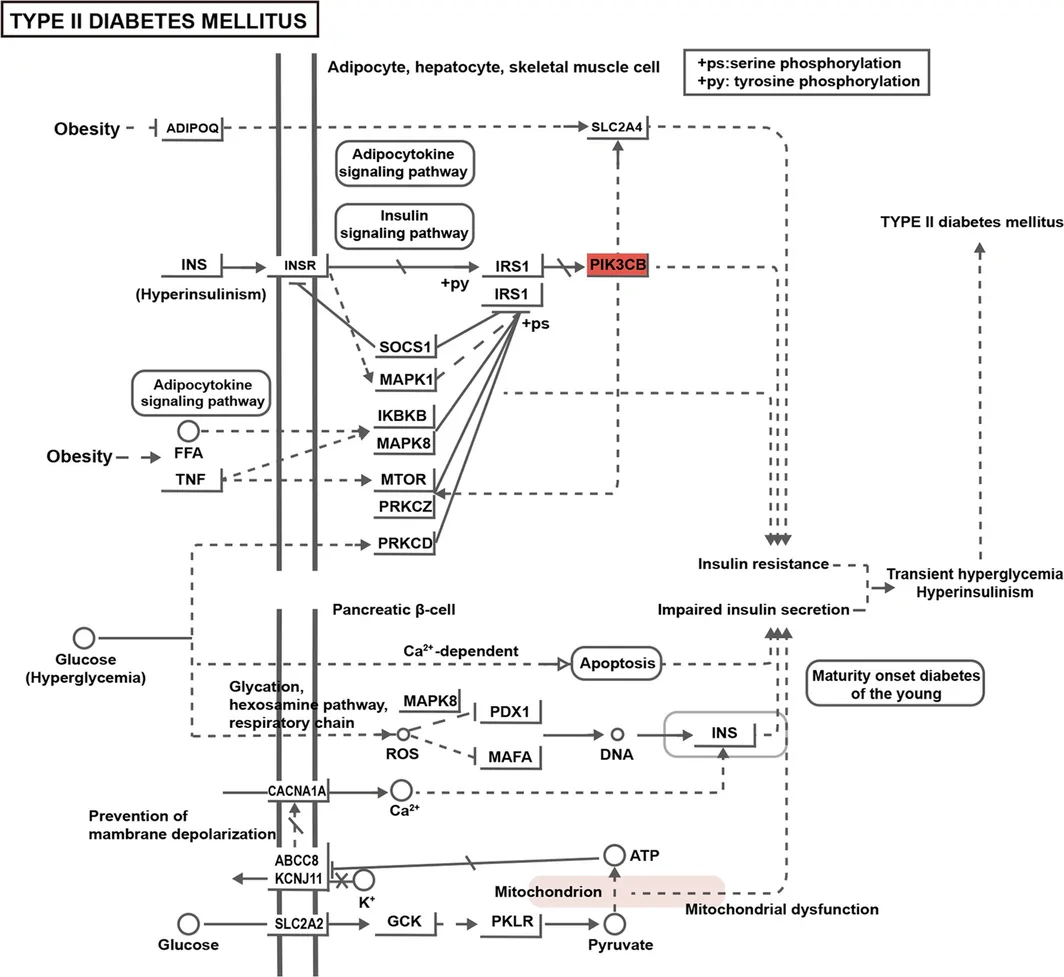
Location of PIK3CB gene in the KEGG pathway.

#### 3.5.2 FHOD1 Gene rs142023201 Site Mutation

FHOD1 is not directly annotated in KEGG glucose metabolism pathways. However, published studies (Kanzaki and Pessin 2001; Lin et al. 2018) have identified alternative mechanisms by which FHOD1 may affect glucose metabolism. The expression product of FHOD1 gene is Formin Homologydomain‐Containing 1 (FHOD1) protein. It has been reported that FHOD1 protein interacts with Insulin regulated aminopeptidase receptor (IRAP). Although IRAP is the main component of GLUT4 vesicles during the glucose transport process, which can promote the ability of GLUT4 in the uptake and transport of glucose. The rs142023201 mutation of the FHOD1 gene led to the substitution of asparagine at the 498th position of its amino acid sequence with serine, thereby inducing the reduced function of FHOD1 protein encoded by FHOD1, the down‐regulated IRAP function, and decreased GLUT4 ability to transport glucose. Therefore, it is speculated that FHOD1 gene mutation may inhibit the glucose uptake and transport process of GLUT4 by suppressing the function of IRAP, giving rise to an increased blood glucose level and the occurrence of DM.

### 3.6 In Silico Functional Prediction and Database Annotation of the Candidate Variants

The in silico functional prediction and database annotation results for the two missense variants are summarized in Table 3. For the EPHX3 p.Glu260Lys variant, SIFT (0.03) and CADD (24.7) predicted deleterious effects, and PolyPhen‐2 (0.503) predicted possible damaging effect, while REVEL (0.178) predicted a neutral effect. The CADD score of 24.7 indicates that this variant ranks among the top 1% most deleterious substitutions in the human genome. In gnomAD v4.1.1, this variant had a global allele frequency of 1.18 × 10^−5^ and was absent in East Asian populations (0/44,890). It is classified in ClinVar as uncertain significance (RCV004115198.2). For the FHOD1 p.Asn498Ser variant, all four prediction tools consistently indicated tolerated/benign effects (SIFT 0.5, PolyPhen‐2 0, CADD 9.086, REVEL 0.061). In gnomAD v4.1.1, this variant had a global allele frequency of 1.92 × 10^−5^ and was also absent in East Asian populations (0/44878). It is classified in ClinVar as likely benign (RCV004924053.1). Queries to the GWAS Catalog returned no results for either variant, and Ensembl phenotype data (release 116) showed no significant associations with any disease or trait beyond the ClinVar entries.

**TABLE 3:** In silico functional prediction and public database annotations of the two candidate missense variants.

	*EPHX3* rs760409070	*FHOD1* rs142023201
Amino acid change	p.Glu260Lys	p.Asn498Ser
SIFT	Deleterious (0.03)	Tolerated (0.5)^a^
PolyPhen‐2	Possibly damaging (0.503)	Benign (0)
CADD (PHRED)	24.7 (deleterious)	9.086 (benign)
REVEL	0.178 (neutral)	0.061 (neutral)
gnomAD v4.1.1 (East Asian)	0/44,890 (0)	0/44,878 (0)
gnomAD v4.1.1 (Global)	19/1,613,900 (1.18 × 10^−5^)	31/1,610,870 (1.92 × 10^−5^)
GWAS Catalog	No results found	No results found
ClinVar classification	Uncertain significance (RCV004115198.2)	Likely benign (RCV004924053.1)

Abbreviations: CADD, Combined Annotation Dependent Depletion; ClinVar, Clinical Significance for Variants relative to Phenotypes database; gnomAD, Genome Aggregation Database; GWAS, Genome‐wide association study; PolyPhen‐2, Polymorphism Phenotyping v2; REVEL, Rare Exome Variant Ensemble Learner; SIFT, Sorting Intolerant From Tolerant.

^a^ SIFT scores for FHOD1 rs142023201 were consistently tolerated across all annotated transcripts (range 0.43–0.5), with the MANE Select transcript (ENST00000258201.9) showing a score of 0.5.

## 4 Discussion

Epoxide hydrolase3 (EPHX3) gene was cloned from complementary DNA (cDNA) by Integrated Molecular Analysis of Gene Expressions (IMAGE) Association in 1999 (Cingolani et al. 2012). It is located in the human chromosome region 19p13.12, and its final coding product is EPHX3 with a molecular weight of 40,909 Da (Arand et al. 1999), which is one kind of EPHX highly expressed in skin, lung, stomach, esophagus, and tongue (Decker et al. 2012). At present, the recognized functions of EPHX mainly include its catalytic effect on epoxide derivatives and its regulatory effect on a variety of biological functions (Edin et al. 2021). In this study, through literature review (Lin et al. 2010), it was found that there was a complex genetic interaction between EPHX3 gene and PIK3CB gene involved in glucose metabolism in the KEGG pathway. EPHX3 gene inhibits the expression of PIK3CB gene, but its specific mechanism remains unclear. PI3K, the expression product of PIK3CB gene, is an important component of PI3K/AKT pathway. It combines with the substrate to activate the downstream signal molecules of PI3K/AKT signaling pathway and realizes the biological regulatory function of PI3K/AKT signaling pathway (Xu et al. 2022). Insulin receptor substrates (IRS) is one of the substrates of PI3K. When insulin acts on the target tissue or organ, the p85 regulatory subunit of PI3K will bind to the corresponding part of substrate IRS, inducing the activation and translocation of the catalytic subunit P110 to cell membrane. P110 can catalyze the phosphorylation of phosphatidylinositol to produce second messengers such as Phosphatidylinositol 4,5‐bisphosphate (PIP2) and Phosphatidylinositol 3,4,5‐Trisphosphate (PIP3). The activated PIP3 will thus bind to the N‐terminal PH domain of AKT to transfer AKT from cytoplasm to cell membrane, and activate the phosphoinositide 3 dependent protein kinase l (PDK1). The activated PDK1 can bind to the AKT phosphorylation site and activate AKT (Kwak and Park 2016; Murthy et al. 2000). Thereafter, the activated AKT acts as the downstream signal molecule of PI3K/AKT signaling pathway on targets such as GLUT4, GSK‐3, and FOXO1, which ultimately reduces the body's blood glucose level by promoting glucose uptake and transport, promoting glycogen synthesis, and inhibiting gluconeogenesis (Alaaeldin et al. 2021; He et al. 2023; Teaney and Cyr 2023). The mutation of EPHX3 gene inhibits the expression of PIK3CB gene; therefore, it is speculated that it may cause insulin resistance, reduce glucose uptake and transport, decrease glycogen synthesis, and increase gluconeogenesis by inhibiting PIK3CB gene and its downstream signaling pathway, leading to an increased blood glucose level in the body, and ultimately causing DM. Consistent with this hypothesis, in silico functional prediction supported a potential deleterious effect of the EPHX3 p.Glu260Lys variant, particularly its high CADD score (≥ 20) placing it among the top 1% most deleterious substitutions in the human genome. Although REVEL predicted a neutral effect, such discordance among tools is not uncommon for extremely rare variants and does not preclude pathogenicity (Kong et al. 2025; Quaio et al. 2022).

Factors that affect insulin signal transduction can be divided into two categories, namely, congenital factors and acquired factors. In the family of this study, the rs760409070 mutation of EPHX3 gene detected belonged to the congenital factor. The 778 “G” base at rs760409070 of EPHX3 gene was replaced with “A” base, as a result, its encoded amino acid sequence was changed, and the glutamic acid at 260 was also changed to lysine, thus partially inducing the inhibition of the expression product of EPHX3 gene. This variant is absent in East Asian populations (gnomAD v4.1.1) and has no prior T2DM associations in ClinVar or the GWAS Catalog, suggesting that its potential role has not been previously recognized. Currently, there have been studies on the relationship between EPHX3 polymorphism and colon cancer (Zhao et al. 2024), laryngeal cancer (Song et al. 2024), and head and neck squamous cell carcinoma (Li et al. 2023), but no research or report regarding the direct relationship between EPHX3 gene mutation and DM, or on this gene mutation in DM families is available at home and abroad. Further functional verification research is still part of the follow‐up work.

Formin homology domain‐containing1 (FHOD1) protein belongs to the methylarginine protein family, and it exists in adipocytes and skeletal muscle cells. FHOD1 protein is a structural protein of fine muscle filaments. Apart from forming fine muscle filaments in muscle cells, it is also detected in a variety of non‐muscle cells and is one of the cytoskeletal proteins. A study on an East Asian (Korean) population found that changes in the methylation level of a specific CpG site (i.e., a DNA methylation site) on the FHOD1 gene were significantly statistically associated with the occurrence of diabetic kidney disease (Kim et al. 2021). Although there is no evidence indicating that FHOD1 directly participates in glucose metabolism leading to DM, one study (Lin et al. 2018) suggests that FHOD1 protein may promote the glucose transport ability of GLUT4 by binding to insulin responsive aminopeptidase (IRAP), thereby improving the utilization of glucose and reducing plasma glucose level. GLUT4 is mainly transferred from the intracellular storage pool to cell membrane in the form of storage vesicles. The increased GLUT4 expression on cell membrane greatly increases the uptake of plasma glucose by skeletal muscle cells and fat cells. Notably, the glucose taken into cells is rapidly phosphorylated to glucose‐6‐phosphate for glycolysis or glycogen synthesis, thereby reducing the blood glucose level in the body (Watson et al. 2004). IRAP is the main component of GLUT4 vesicles, which contains two di‐leucine motifs and multiple GLUT4‐like domains that play important roles in regulating the transport of GLUT4 vesicles (Sui and Guo 2021). In addition, Tojo et al. (2003) found that FHOD1 protein interacted with actin‐binding fibrin, resulting in the increased intracellular insulin‐stimulated glucose uptake, but the overexpression of FHOD1 that lacked the IRAP binding domain did not promote insulin‐stimulated glucose uptake.

The rs142023201 mutation of the FHOD1 gene leads to the change in the amino acid sequence encoded by the FHOD1 gene; to be specific, Asparagine at position 498 is replaced by serine, which results in the decrease in FHOD1 protein function and causes damage to the glucose transport process jointly participated in by IRAP and GLUT4, finally leading to DM (Kanzaki and Pessin 2001). Consistently, public database queries revealed no prior evidence linking this variant to T2DM: it is absent in East Asian populations, classified as likely benign in ClinVar, and has no recorded associations in the GWAS Catalog. At present, most studies on FHOD1 gene polymorphism at home and abroad have focused on hypertrophic cardiomyopathy (García Hernandez et al. 2025; Piqueras‐Flores et al. 2024) and leukemia (French et al. 2009), but there is no study concerning the direct correlation between FHOD1 gene mutation and the occurrence of DM, or on this gene mutation in the DM family. Notably, in silico predictions for the FHOD1 p.Asn498Ser variant consistently indicated no deleterious effect across all four tools. However, current computational tools are primarily trained on sequence conservation and may perform poorly for structural/cytoskeletal proteins like FHOD1, where function depends on protein–protein interactions rather than enzymatic activity (Gasteier et al. 2003; Porollo and Meller 2007). Therefore, the negative in silico predictions do not negate the family‐based co‐segregation evidence. Functional studies like assessing IRAP binding or GLUT4 translocation are needed to determine the actual impact of this variant.

In this study, the heterozygous mutations of EPHX3 gene rs760409070 [c.778 G>A (p.Glu260Lys)] and FHOD1 gene rs142023201 [c.1493 A>G (p.Asn498Ser)] were found to be related to the occurrence of DM. However, it remains unclear whether these two mutations act independently, synergistically, or whether one is a primary driver and the other a secondary modifier, which requires further functional studies (e.g., cell‐based assays evaluating PI3K/AKT activity or GLUT4 translocation) and experimental validation. These two mutations were present in all five diabetic patients in the family but absent in the second‐generation normal controls, and their occurrence frequency in the third‐generation direct relatives was less than 50%. Although the third‐generation relatives are currently young and have not yet been diagnosed with DM, they may be at increased risk. Therefore, it is recommended that third‐generation direct relatives in this family, especially those carrying either the EPHX3 rs760409070 heterozygous mutation or the FHOD1 rs142023201 heterozygous mutation, adopt a healthy lifestyle as soon as possible to reduce or delay the onset of DM. This also provides a basis for follow‐up studies and functional verification.

Yunnan Province is characterized by high ethnic diversity and distinct genetic architecture (Wang et al. 2025). Previous genetic studies on T2DM in Yunnan populations have identified associations with genes such as MTHFR (Liu et al. 2024), ARL15 (Li et al. 2014), CDH13 (Li et al. 2017), RANTES and CCR5 (Chen et al. 2023). To our knowledge, no prior studies have reported EPHX3 or FHOD1 variants in any Yunnan population with T2DM. This gap is consistent with the absence of both variants in East Asian populations, reinforcing their rarity and the lack of prior recognition. Our findings thus add novel candidate genes to the regional genetic landscape. However, large‐scale population genomic data for Yunnan remain limited, and we cannot determine whether these variants are specific to this pedigree or represent broader regional risk factors. Future studies with larger Yunnan cohorts are needed to assess the generalizability of our findings.

This study has several limitations. First, the findings from a single pedigree require validation in larger cohorts and sporadic diabetic populations to confirm generalizability. Second, potential environmental modifiers (e.g., dietary habits, physical activity) that may influence disease expression were not assessed, and BMI data for non‐diabetic family members were also unavailable, which could contribute to the incomplete penetrance or variable expressivity of the identified variants. Third, we cannot definitively determine whether the EPHX3 or FHOD1 mutation is individually causal or whether they act together; Functional studies (e.g., overexpression or knockdown in insulin‐responsive cell lines) are needed to assess their effects on insulin signaling and glucose transport. Fourth, detailed insulin resistance or secretion indices (e.g., Homeostatic Model Assessment for Insulin Resistance [HOMA‐IR], C‐peptide) were not available for most patients due to the retrospective nature of data collection, limiting genotype–phenotype correlations; moreover, the absence of such data in non‐diabetic family members carrying the identified variants precludes assessment of the variants' primary physiological effects independent of hyperglycemia. These limitations underscore the need for comprehensive clinical profiling in future studies.

## 5 Conclusion

Through whole exome sequencing and bioinformatic analysis, it is found that the mutations of EPHX3 gene rs760409070[c.778 G>A (p.Glu260Lys)] and FHOD1 gene rs142023201[c.1493 A>G (p.Asn498Ser)] may be the pathogenic genes and mutation sites of T2DM among the family members. These two mutations may contribute to the development of DM by reducing peripheral tissue sensitivity to insulin, decreasing glucose uptake, inhibiting glycogen synthesis, and promoting gluconeogenesis.

## Author Contributions

**Jie Chen:** conceptualization, writing – original draft, methodology, formal analysis, software, investigation, data curation. **Jia‐Li Li:** conceptualization, writing – original draft, investigation, methodology, software, formal analysis, data curation. **He‐Jia Yin:** data curation, investigation, conceptualization, methodology. **Wei‐Chang Guo:** investigation, data curation, conceptualization, methodology. **Hui‐Fang Li:** conceptualization, investigation, funding acquisition, writing – review and editing, methodology, supervision, project administration, validation.

## Funding

This work was supported by National Natural Science Foundation of China, 30760087. The “Famous Doctor” Special Program of Yunnan Xingdian Talent Support Plan, RLMY20220009. The Young and Middle‐aged Academic and Technical Leaders Reserve Talents Project of Yunnan Province, 202105AC160093. The Key Project of Yunnan Provincial Clinical Medical Center for Endocrine and Metabolic Diseases, 2024YNLCYXZX0073.

## Conflicts of Interest

The authors declare no conflicts of interest.

## Supporting information


**Table S1:** Clinical characteristics of affected family members with T2DM.

## Data Availability

The data that support the findings of this study are available from the corresponding author upon reasonable request.

## References

[mgg370296-bib-0001] Alaaeldin, R. , I. A. M. Abdel‐Rahman , H. A. Hassan , et al. 2021. “Carpachromene Ameliorates Insulin Resistance in HepG2 Cells via Modulating IR/IRS1/PI3k/Akt/GSK3/FoxO1 Pathway.” Molecules 26, no. 24: 7629. 10.3390/molecules26247629. PMC8708443 34946711

[mgg370296-bib-0002] Arand, M. , H. Hemmer , H. Dürk , et al. 1999. “Cloning and Molecular Characterization of a Soluble Epoxide Hydrolase From Aspergillus Niger That Is Related to Mammalian Microsomal Epoxide Hydrolase.” Biochemical Journal 344, no. Pt 1: 273–280. 10.1042/0264-6021:3440273. PMC1220641 10548561

[mgg370296-bib-0003] Asamoah, E. A. , C. Obirikorang , E. Acheampong , et al. 2020. “Heritability and Genetics of Type 2 Diabetes Mellitus in Sub‐Saharan Africa: A Systematic Review and Meta‐Analysis.” Journal of Diabetes Research 2020: 3198671. 10.1155/2020/3198671. PMC7352126 32685554

[mgg370296-bib-0004] Benrahma, H. , I. Arfa , M. Charif , et al. 2011. “Maternal Effect and Familial Aggregation in a Type 2 Diabetic Moroccan Population.” Journal of Community Health 36, no. 6: 943–948. 10.1007/s10900-011-9393-3. 21442339

[mgg370296-bib-0005] Chen, J. , W. Guo , H. Yin , L. Ma , S. Li , and H. Li . 2023. “Investigation of the Correlation Between the Polymorphism/Expression Level of RANTES and Its Receptor CCR5 Gene Promoter and Type 2 Diabetes Mellitus.” Diabetes, Metabolic Syndrome and Obesity: Targets and Therapy 16: 213–223. 10.2147/dmso.S398264. PMC9884060 36760585

[mgg370296-bib-0006] Cingolani, P. , A. Platts , L. Wang le , et al. 2012. “A Program for Annotating and Predicting the Effects of Single Nucleotide Polymorphisms, SnpEff: SNPs in the Genome of *Drosophila melanogaster* Strain w1118; Iso‐2; Iso‐3.” Fly (Austin) 6, no. 2: 80–92. 10.4161/fly.19695. PMC3679285 22728672

[mgg370296-bib-0007] Decker, M. , M. Adamska , A. Cronin , et al. 2012. “EH3 (ABHD9): The First Member of a New Epoxide Hydrolase Family With High Activity for Fatty Acid Epoxides.” Journal of Lipid Research 53, no. 10: 2038–2045. 10.1194/jlr.M024448. PMC3435537 22798687

[mgg370296-bib-0008] Deutsch, A. J. , and M. S. Udler . 2025. “Phenotypic and Genetic Diversity in Diabetes Across Populations.” Journal of Clinical Endocrinology and Metabolism 110, no. 8: 2123–2133. 10.1210/clinem/dgaf234. PMC12641533 40231339

[mgg370296-bib-0009] Edin, M. L. , H. Yamanashi , W. E. Boeglin , et al. 2021. “Epoxide Hydrolase 3 (Ephx3) Gene Disruption Reduces Ceramide Linoleate Epoxide Hydrolysis and Impairs Skin Barrier Function.” Journal of Biological Chemistry 296: 100,198. 10.1074/jbc.RA120.016570. PMC7948417 33334892

[mgg370296-bib-0010] French, D. , W. Yang , C. Cheng , et al. 2009. “Acquired Variation Outweighs Inherited Variation in Whole Genome Analysis of Methotrexate Polyglutamate Accumulation in Leukemia.” Blood 113, no. 19: 4512–4520. 10.1182/blood-2008-07-172,106. PMC2680361 19066393

[mgg370296-bib-0011] García Hernandez, S. , L. de la Higuera Romero , A. Fernandez , et al. 2025. “Redefining the Genetic Architecture of Hypertrophic Cardiomyopathy: Role of Intermediate‐Effect Variants.” Circulation 152, no. 15: 1060–1075. 10.1161/circulationaha.125.074529. PMC12513046 40879562

[mgg370296-bib-0012] Gasteier, J. E. , R. Madrid , E. Krautkrämer , et al. 2003. “Activation of the Rac‐Binding Partner FHOD1 Induces Actin Stress Fibers via a ROCK‐Dependent Mechanism.” Journal of Biological Chemistry 278, no. 40: 38902–38912. 10.1074/jbc.M306229200. 12857739

[mgg370296-bib-0013] Halldorsson, B. V. , H. P. Eggertsson , K. H. S. Moore , et al. 2022. “The Sequences of 150,119 Genomes in the UK Biobank.” Nature 607, no. 7920: 732–740. 10.1038/s41586-022-04965-x. PMC9329122 35859178

[mgg370296-bib-0014] He, C. , K. Wang , J. Xia , et al. 2023. “Natural Exosomes‐Like Nanoparticles in Mung Bean Sprouts Possesses Anti‐Diabetic Effects via Activation of PI3K/Akt/GLUT4/GSK‐3β Signaling Pathway.” Journal of Nanobiotechnology 21, no. 1: 349. 10.1186/s12951-023-02120-w. PMC10536756 37759297

[mgg370296-bib-0015] Huerta‐Chagoya, A. , P. Schroeder , R. Mandla , et al. 2024. “Rare Variant Analyses in 51,256 Type 2 Diabetes Cases and 370,487 Controls Reveal the Pathogenicity Spectrum of Monogenic Diabetes Genes.” Nature Genetics 56, no. 11: 2370–2379. 10.1038/s41588-024-01947-9. PMC11549050 39379762

[mgg370296-bib-0016] Kalyani, R. R. , J. J. Neumiller , N. M. Maruthur , and D. J. Wexler . 2025. “Diagnosis and Treatment of Type 2 Diabetes in Adults: A Review.” JAMA 334, no. 11: 984–1002. 10.1001/jama.2025.5956. PMC13014273 40549398

[mgg370296-bib-0017] Kanehisa, M. , M. Furumichi , Y. Sato , M. Kawashima , and M. Ishiguro‐Watanabe . 2023. “KEGG for Taxonomy‐Based Analysis of Pathways and Genomes.” Nucleic Acids Research 51, no. D1: D587–D592. 10.1093/nar/gkac963. PMC9825424 36300620

[mgg370296-bib-0018] Kanzaki, M. , and J. E. Pessin . 2001. “Insulin‐Stimulated GLUT4 Translocation in Adipocytes Is Dependent Upon Cortical Actin Remodeling.” Journal of Biological Chemistry 276, no. 45: 42,436–42,444. 10.1074/jbc.M108297200. 11546823

[mgg370296-bib-0019] Karczewski, K. J. , B. Weisburd , B. Thomas , et al. 2017. “The ExAC Browser: Displaying Reference Data Information From Over 60,000 Exomes.” Nucleic Acids Research 45, no. D1: D840–D845. 10.1093/nar/gkw971. PMC5210650 27899611

[mgg370296-bib-0020] Kim, H. , J. H. Bae , K. S. Park , J. Sung , and S. H. Kwak . 2021. “DNA Methylation Changes Associated With Type 2 Diabetes and Diabetic K Idney Disease in an East Asian Population.” Journal of Clinical Endocrinology and Metabolism 106, no. 10: e3837–e3851. 10.1210/clinem/dgab488. 34214161

[mgg370296-bib-0021] Kong, S. W. , I. H. Lee , L. V. Collen , et al. 2025. “Discordance Between a Deep Learning Model and Clinical‐Grade Variant Pathogenicity Classification in a Rare Disease Cohort.” NPJ Genomic Medicine 10, no. 1: 17. 10.1038/s41525-025-00480-w. PMC11871343 40021654

[mgg370296-bib-0022] Kwak, S. H. , and K. S. Park . 2016. “Recent Progress in Genetic and Epigenetic Research on Type 2 Diabetes.” Experimental & Molecular Medicine 48, no. 3: e220. 10.1038/emm.2016.7. PMC4892885 26964836

[mgg370296-bib-0023] Lek, M. , K. J. Karczewski , E. V. Minikel , et al. 2016. “Analysis of Protein‐Coding Genetic Variation in 60,706 Humans.” Nature 536, no. 7616: 285–291. 10.1038/nature19057. PMC5018207 27535533

[mgg370296-bib-0024] Li, Y. , C. Li , Y. Yang , et al. 2017. “The Association of Six Single Nucleotide Polymorphisms and Their Haplotypes in CDH13 With T2DM in a Han Chinese Population.” Medicine (Baltimore) 96, no. 22: e7063. 10.1097/md.0000000000007063. PMC5459737 28562572

[mgg370296-bib-0025] Li, Y. , Y. Yang , Y. Yao , et al. 2014. “Association Study of ARL15 and CDH13 With T2DM in a Han Chinese Population.” International Journal of Medical Sciences 11, no. 5: 522–527. 10.7150/ijms.8206. PMC3970107 24688318

[mgg370296-bib-0026] Li, Z. , H. Cai , J. Zheng , et al. 2023. “Mitochondrial‐Related Genes Markers That Predict Survival in Patients With Head and Neck Squamous Cell Carcinoma Affect Immunomodulation Through Hypoxia, Glycolysis, and Angiogenesis Pathways.” Aging 15, no. 19: 10,347–10,369. 10.18632/aging.205081. PMC10599748 37796226

[mgg370296-bib-0027] Liao, P. J. , M. K. Ting , C. F. Kuo , Y. H. Ding , C. M. Lin , and K. H. Hsu . 2023. “Kinship Analysis of Type 2 Diabetes Mellitus Familial Aggregation in Taiwan.” Biomedical Journal 46, no. 4: 100,549. 10.1016/j.bj.2022.07.003. PMC10345230 35863666

[mgg370296-bib-0028] Lin, A. , R. T. Wang , S. Ahn , C. C. Park , and D. J. Smith . 2010. “A Genome‐Wide Map of Human Genetic Interactions Inferred From Radiation Hybrid Genotypes.” Genome Research 20, no. 8: 1122–1132. 10.1101/g.104216.109. PMC2909575 20508145

[mgg370296-bib-0029] Lin, X. , H. Shi , Y. Cui , et al. 2018. “Dendrobium Mixture Regulates Hepatic Gluconeogenesis in Diabetic Rats via the Phosphoinositide‐3‐Kinase/Protein Kinase B Signaling Pathway.” Experimental and Therapeutic Medicine 16, no. 1: 204–212. 10.3892/etm.2018.6194. PMC5995077 29896241

[mgg370296-bib-0030] Liu, Y. , G. Pu , C. Yang , et al. 2024. “Association Analysis of MTHFR (rs1801133 and rs1801131) Gene Polymorphism Towards the Development of Type 2 Diabetes Mellitus in Dali Area Population From Yunnan Province, China.” PeerJ 12: e18334. 10.7717/peerj.18334. PMC11512809 39465169

[mgg370296-bib-0031] Montojo, J. , K. Zuberi , H. Rodriguez , et al. 2010. “GeneMANIA Cytoscape Plugin: Fast Gene Function Predictions on the Desktop.” Bioinformatics 26, no. 22: 2927–2928. 10.1093/bioinformatics/btq562. PMC2971582 20926419

[mgg370296-bib-0032] Murthy, S. S. , A. Tosolini , T. Taguchi , and J. R. Testa . 2000. “Mapping of AKT3, Encoding a Member of the AKT/Protein Kinase B Family, to Human and Rodent Chromosomes by Fluorescence In Situ Hybridization.” Cytogenetics and Cell Genetics 88, no. 1–2: 38–40. 10.1159/000015481. 10773662

[mgg370296-bib-0033] Piqueras‐Flores, J. , E. Villacorta‐Argüelles , J. Galvin , et al. 2024. “Intermediate‐Effect Size p.Arg637Gln in FHOD3 Increases Risk of HCM and Is Associated With an Aggressive Phenotype in Homozygous Carriers.” Journal of Medical Genetics 61, no. 5: 423–427. 10.1136/jmg-2023-109,413. 38160043

[mgg370296-bib-0034] Porollo, A. , and J. Meller . 2007. “Prediction‐Based Fingerprints of Protein–Protein Interactions.” Proteins 66, no. 3: 630–645. 10.1002/prot.21248. 17152079

[mgg370296-bib-0035] Quaio, C. C. , J. R. M. Ceroni , M. C. Cervato , et al. 2022. “Parental Segregation Study Reveals Rare Benign and Likely Benign Variants in a Brazilian Cohort of Rare Diseases.” Scientific Reports 12, no. 1: 7764. 10.1038/s41598-022-11,932-z. PMC9095660 35546177

[mgg370296-bib-0036] Sherry, S. T. , M. H. Ward , M. Kholodov , et al. 2001. “dbSNP: The NCBI Database of Genetic Variation.” Nucleic Acids Research 29, no. 1: 308–311. 10.1093/nar/29.1.308. PMC29783 11125122

[mgg370296-bib-0037] Shojima, N. , and T. Yamauchi . 2023. “Progress in Genetics of Type 2 Diabetes and Diabetic Complications.” Journal of Diabetes Investigation 14, no. 4: 503–515. 10.1111/jdi.13970. PMC10034958 36639962

[mgg370296-bib-0038] Song, D. , L. Dong , M. Wang , and X. Gao . 2024. “Identification of a Novel Five‐Gene Prognostic Model for Laryngeal Cancer Associated With Mitophagy Using Integrated Bioinformatics Analysis and Experimental Verification.” Critical Reviews in Immunology 44, no. 6: 75–85. 10.1615/CritRevImmunol.2024051787. 38848295

[mgg370296-bib-0039] Sui, L. , and H. C. Guo . 2021. “Enhanced Recombinant Expression and Purification of Human IRAP for Biochemical and Crystallography Studies.” Biochemistry and Biophysics Reports 27: 101042. 10.1016/j.bbrep.2021.101042. PMC8207215 34169156

[mgg370296-bib-0040] Sun, H. , P. Saeedi , S. Karuranga , et al. 2022. “IDF Diabetes Atlas: Global, Regional and Country‐Level Diabetes Prevalence Estimates for 2021 and Projections for 2045.” Diabetes Research and Clinical Practice 183: 109119. 10.1016/j.diabres.2021.109119. PMC11057359 34879977

[mgg370296-bib-0041] Suzuki, K. , K. Hatzikotoulas , L. Southam , et al. 2024. “Genetic Drivers of Heterogeneity in Type 2 Diabetes Pathophysiology.” Nature 627, no. 8003: 347–357. 10.1038/s41586-024-07019-6. PMC10937372 38374256

[mgg370296-bib-0042] Tam, C. H. , Y. Wang , J. Luan , et al. 2014. “Maternal History of Diabetes Is Associated With Increased Cardiometabolic Risk in Chinese.” Nutrition & Diabetes 4, no. 3: e112. 10.1038/nutd.2014.9. PMC3974036 24614663

[mgg370296-bib-0043] Tangjittipokin, W. , T. Narkdontri , N. Teerawattanapong , et al. 2025. “Investigation of the Degree of Family History of Diabetes in Different Clusters of Newly Diagnosed Type 2 Diabetes in Thailand.” Annals of Medicine 57, no. 1: 2500697. 10.1080/07853890.2025.2500697. PMC12064116 40338056

[mgg370296-bib-0044] Teaney, N. A. , and N. E. Cyr . 2023. “FoxO1 as a Tissue‐Specific Therapeutic Target for Type 2 Diabetes.” Frontiers in Endocrinology 14: 1286838. 10.3389/fendo.2023.1286838. PMC10629996 37941908

[mgg370296-bib-0045] Tojo, H. , I. Kaieda , H. Hattori , et al. 2003. “The Formin Family Protein, Formin Homolog Overexpressed in Spleen, Interacts With the Insulin‐Responsive Aminopeptidase and Profilin IIa.” Molecular Endocrinology 17, no. 7: 1216–1229. 10.1210/me.2003-0056. 12677009

[mgg370296-bib-0046] Walker, J. T. , D. C. Saunders , V. Rai , et al. 2023. “Genetic Risk Converges on Regulatory Networks Mediating Early Type 2 Diabetes.” Nature 624, no. 7992: 621–629. 10.1038/s41586-023-06693-2. PMC11374460 38049589

[mgg370296-bib-0047] Wang, T. , M. A. Yang , Z. Zhu , et al. 2025. “Prehistoric Genomes From Yunnan Reveal Ancestry Related to Tibetans and Austroasiatic Speakers.” Science 388, no. 6750: eadq9792. 10.1126/science.adq9792. 40440384

[mgg370296-bib-0048] Watson, R. T. , M. Kanzaki , and J. E. Pessin . 2004. “Regulated Membrane Trafficking of the Insulin‐Responsive Glucose Transporter 4 in Adipocytes.” Endocrine Reviews 25, no. 2: 177–204. 10.1210/er.2003-0011. 15082519

[mgg370296-bib-0049] Wei, J. N. , H. Y. Li , Y. C. Wang , et al. 2010. “Detailed Family History of Diabetes Identified Children at Risk of Type 2 Diabetes: A Population‐Based Case–Control Study.” Pediatric Diabetes 11, no. 4: 258–264. 10.1111/j.1399-5448.2009.00564.x. 19708906

[mgg370296-bib-0050] Xu, W. , Z. Wang , Z. Zhang , J. Xu , and Y. Jiang . 2022. “PIK3CB Promotes Oesophageal Cancer Proliferation Through the PI3K/AKT/mTOR Signalling Axis.” Cell Biology International 46, no. 9: 1399–1408. 10.1002/cbin.11847. 35842767

[mgg370296-bib-0051] Zhao, B. , J. Wang , G. Sheng , Y. Wang , T. Yang , and K. Meng . 2024. “Identifying a Risk Signature of Methylation‐Driven Genes as a Predictor of Survival Outcome for Colon Cancer Patients.” Applied Biochemistry and Biotechnology 196, no. 7: 4156–4165. 10.1007/s12010-023-04751-z. 37906409

