# Supplementary material for: Analysis of Whole Exome Sequencing Results of Type 2 Diabetic Pedigree
Source: Mol Genet Genomic Med. 2026 Oct 21;14(10):e70296. doi: 10.1002/mgg3.70296 (PMC13685523; doi:10.1002/mgg3.70296)
Supplement: Supplementary file 1 [file MGG3-14-e70296-s001.docx]

**Supplementary Table S1. Clinical characteristics of affected family members with T2DM**

| **ID** | **Age (years)** | **Age at diagnosis (years)** | **Height (cm)** | **Weight (kg)** | **BMI (kg/m²)** | **HbA1c (%)** | **FBG (mmol/L)** | **2h PBG (mmol/L)** | **Treatment** | **Complications** |
| --- | --- | --- | --- | --- | --- | --- | --- | --- | --- | --- |
| G1 | 76 | 40 | 160 | 78 | 30.47 | 11.0 | 13.5 | 15.4 | Insulin | Diabetic nephropathy, diabetic retinopathy |
| G2 | 55 | 45 | 162 | 70 | 26.67 | 9.0 | 11.1 | 12.4 | Metformin + acarbose | Diabetic peripheral neuropathy |
| G4 | 52 | 46 | 164 | 75 | 27.89 | 8.0 | 12.0 | 14.3 | Metformin | Diabetic peripheral neuropathy |
| G5 | 48 | 42 | 161 | 73 | 28.16 | 9.2 | 10.4 | 12.0 | Insulin | Diabetic peripheral neuropathy |
| G7 | 45 | 43 | 163 | 68 | 25.59 | 7.0 | 9.0 | 10.5 | Metformin | Diabetic peripheral neuropathy |

**Notes:** FBG, fasting blood glucose; 2h PBG, 2-hour postprandial blood glucose; BMI, body mass index; HbA1c, glycated hemoglobin.
